# Analyzing the heterogeneity of labor and delivery units: A quantitative analysis of space and design

**DOI:** 10.1371/journal.pone.0209339

**Published:** 2018-12-26

**Authors:** Naola Austin, Alexandria Kristensen-Cabrera, Jules Sherman, Doug Schwandt, Allison McDonald, Laura Hedli, Lillian Sie, Steven Lipman, Kay Daniels, Lou P. Halamek, Henry C. Lee

**Affiliations:** 1 Department of Anesthesiology, Perioperative and Pain Medicine, School of Medicine, Stanford University, Palo Alto, California, United States of America; 2 Department of Pediatrics, Neonatal and Developmental Medicine, School of Medicine, Stanford University, Palo Alto, California, United States of America; 3 Department of Obstetrics & Gynecology, Maternal Fetal Medicine, School of Medicine, Stanford University, Palo Alto, California, United States of America; Case Western Reserve University, UNITED STATES

## Abstract

This study assessed labor and delivery (L&D) unit space and design, and also considered correlations between physical space measurements and clinical outcomes. Design and human factors research has increased standardization in high-hazard industries, but is not fully utilized in medicine. Emergency department and intensive care unit space has been studied, but optimal L&D unit design is undefined. In this prospective, observational study, a multidisciplinary team assessed physical characteristics of ten L&D units. Design measurements were analyzed with California Maternal Quality Care Collaborative (CMQCC) data from 34,161 deliveries at these hospitals. The hospitals ranged in delivery volumes (<1000–>5000 annual deliveries) and cesarean section rates (19.6%-39.7%). Within and among units there was significant heterogeneity in labor room (LR) and operating room (OR) size, count, and number of configurations. There was significant homogeneity of room equipment. Delivery volumes correlated with unit size, room counts, and cesarean delivery rates. Relative risk of cesarean section was modestly increased when certain variables were above average (delivery volume, unit size, LR count, OR count, OR configuration count, LR to OR distance, unit utilization) or below average (LR size, OR size, LR configuration count). Existing variation suggests a gold standard design has yet to be adopted for L&D. A design-centered approach identified opportunities for standardization: 1) L&D unit size and 2) room counts based on current or projected delivery volume, and 3) LR and OR size and equipment. When combined with further human factors research, these guidelines could help design the L&D unit of the future.

## Introduction

Design standardization is fundamental to safety in high-stakes industries, such as aviation, aeronautics, and nuclear power [1]. Standardization efforts in medicine [2, 3] and toward reducing severe maternal morbidity [4–9] tend to discuss specific problems, equipment, or processes. With limited exceptions [10], medicine is arguably behind other high-hazard industries in devising guidelines on physical design aimed to improve safety and outcomes [11, 12]. Standardization of systems in obstetrics may reduce medical and medicolegal complications [13, 14].

Patient care on labor and delivery (L&D) is complex and presents several design challenges. An optimally designed unit would maximize safety, efficiency, and accommodate the unpredictable timing and sudden surges in acuity, and volume inherent to maternal and perinatal medicine. Designs are subject to multiple influences such as building codes, available guidelines [15–19], budget constraints, and input from various stakeholders (i.e., architects, builders, engineers, executives, clinicians, and patients). Providers and patients who best understand the user experience have variable involvement with managers, executives and other professionals responsible for designing the unit.

Design and human factors research has been extensively applied to cockpits and kitchens, but studies that apply design thinking (i.e., inspiration, ideation, implementation) to medical domains are relatively rare. While better understood in other healthcare settings [20–26], the impact of physical design on clinical outcomes on L&D is of growing interest [14, 27] and facilities factors may play an important role in patient safety [28, 29].

Little research exists on the design heterogeneity among different L&D units and the best practices for L&D unit design are undefined. Utilizing design thinking, we sought to understand and quantify space and design of existing L&D units. We aimed to 1) evaluate the physical space, and 2) inventory and measure equipment, and 3) correlate measurements of L&D unit design with delivery volume and basic clinical outcomes of vaginal and cesarean birth.

## Materials and methods

Between July 2015 and February 2017, ten academic and private labor and delivery units in California were measured in this observational study approved by the Stanford University Institutional Review Board (IRB). Thirteen units were invited to participate via phone, email, or in-person invitation, and the first ten units that accepted the invitation were included in the study. Institutional refusal was the only exclusion criteria. Local providers were present during the visit and were given a written study description and a copy of the IRB study information.

Our interdisciplinary team included clinicians as well as a design expert, mechanical engineer, architect, and other researchers. The clinicians included anesthesiologists, obstetricians and neonatologists. Design expert JPS visited all 10 sites; she is medical product designer and lecturer at the Hasso Plattner Institute of Design at Stanford, and architect RM has experience in project design of large, academic hospitals. Our team also had expertise in quality improvement. Neonatologist and Co-PI of our Safety Learning Laboratory, HCL, is also the Chief Medical Officer for the California Perinatal Quality Care Collaborative (CPQCC). The CPQCC is a statewide quality improvement initiative with 137 member hospitals (to-date), accounting for over 90 percent of all infants cared for in California NICUs. Dr. Lee has been a member of the Perinatal Quality Improvement Panel within CPQCC since 2009.

With the assistance of providers at each institution, members of our team toured facilities and acquired direct measurements of labor rooms, operating rooms and other areas as well as equipment within the rooms. We limited our study to analysis of the labor and delivery unit only. Other locations in the hospital whose proximity might be relevant to L&D (NICU, Adult Main Operating Room, Pediatric Main Operating Rooms, Interventional Radiology, Emergency Room, Radiology) were numerous and deemed outside the scope of our primary research aim. In this paper, we have provided a more thorough discussion of how units are designed based on building codes and guidelines for the planning, design and construction of hospitals. While we did not factor in the guidelines outlined by the American College of Healthcare Architects (ACHA) into our analysis, an architect member of the research group (RM) is a member of American Institute of Architects.

Direct measurements and photographs were combined with facility architectural maps and evaluated with computer-aided design software (Dassault Systemes SolidWorks Corporation, Waltham, MA) to maximize accuracy. Irrespective of the volume each hospital was initially designed to accommodate, current physical design measurements were then evaluated in the context of 2015 hospital delivery data from the California Maternal Quality Care Collaborative (CMQCC), CPQCC’s sister organization. This state-wide quality improvement initiative collects near real-time data on over 200 hospitals, based on patient discharge data from the Office of Statewide Health Planning and Development which is then linked to birth certificate data from the California Department of Public Health-Vital Records. We were specifically interested in total and NTSV cesarean rates. Details of these individual measurements and comparisons are explained with the results.

We conducted descriptive statistical analysis to examine design characteristics (i.e., mean distances and standard deviations (SDs) and delivery volumes and rates across our sample. Friedman’s ANOVA was calculated to compare mean labor room to operating room distances between different labor units. Pearson correlation coefficients were calculated to assess the relationship between room counts and both delivery volumes and rates. Data were analyzed using SAS v24.

Data were stored on secure platforms and results are presented with intention to preserve institutional anonymity as much as possible while still allowing for comparisons by hospital characteristics. Patient privacy was prioritized, and photographs or direct measurements in the presence of patients or protected health information was strictly avoided.

## Results

### Demographics and unit characteristics

The combined annual delivery volume for the ten labor units was 34,161. We were unable to determine the exact number of annual deliveries that the units were originally designed to accommodate. Instead, we measured the current number of annual deliveries with the existing design.

The distribution of 2015 delivery volumes and rates is shown in Table 1. Percentage of total deliveries that were cesarean deliveries within an institution (cesarean delivery rate) ranged from 19.6% to 39.7% (mean 29.0%, *SD* 5.0%). Percentage of nulliparous, term, singleton, vertex (NTSV) deliveries that were cesarean deliveries (NTSV cesarean rate) ranged from 14.8% to 32.5% (mean 23.7%, *SD* 4.8%). The vast majority of scheduled and unscheduled deliveries were performed on the units we reviewed. Rare, very complex cesarean deliveries were performed off of L&D (i.e. certain abnormal placentation, ex utero intrapartum treatment procedure, fetal surgery, etc.).

**Table 1:** Hospital characteristics.

**Annual Delivery Volume**	**Number of Hospitals**
<1000	1
1000–2000	2
2000–3000	3
4000–5000	3
>5000	1
**Delivery Volume or Rate**	**Mean (SD)**
Annual Deliveries	3416 (2490)
Annual Vaginal Deliveries	2326 (1399)
Annual Cesarean Deliveries	1090 (1029)
Cesarean Delivery Rate	29.0% (5.0%)
NTSV Cesarean Rate	23.7% (4.8%)
**Maternal Level of Care**[30]	**Number of Hospitals**
Birth Center	0
I: Basic Care	0
II: Specialty Care	2
III: Sub-specialty Care	5
IV: Regional Perinatal Center	3
**NICU Level**[31]	**Number of Hospitals**
I: Basic Care	0
II: Specialty Care	2
III: Sub-specialty Care	6
IV: III with Additional Capabilities	2
**District Type**[32]	**Number of Hospitals**
Urban (City: Large)	8
Suburban (Suburb: Large)	2
Rural	0
**Hospital Type**[33]	**Number of Hospitals**
Private, non-profit	8
Public, county owned	1
Public, university owned	1
**Payer Type**	**% of patients**
Medi-Cal	21.7%
Commercial Insurance	73.3%
Self-Pay	2.3%
Other	2.8%

Cesarean delivery rate: number of cesarean deliveries out of total deliveries per 100 live births. NTSV Cesarean Delivery Rate: Nulliparous, term, singleton, vertex (NTSV) cesarean deliveries out of total NTSV deliveries. NICU: Neonatal Intensive Care Unit. Maternal Level of Care defined by the American College of Obstetricians and Gynecologists and the Society for Maternal-Fetal Medicine. NICU Level of Care defined by the American Academy of Pediatrics. District Type defined by the National Center for Education Statistics. Hospital Type defined by the California Department of Public Health

We were unable to accurately estimate and report the age of each unit visited. Some L&D units existed in buildings that were very old (the oldest building we visited being erected in the 1870s), where different parts had been remodeled at different times. We observed several examples of how original construction and remodels were impacted by building codes that changed every several years. For example, the L&D unit at our home institution first opened in 1991. The labor and operating rooms were built based on the building codes in existence at that time. A third operating room was built in approximately 2000. Because building codes changed between when the hospital was first built and when the third operating room was added, the third operating room was larger than the first two. While the current configuration of this unit has remained the same since the early 2000s, the majority of the unit is nearly 30 years old. Therefore, in this example, it would inaccurate to report the age of the unit as 15 or 30 years old.

Most labor and operating rooms were located on the same floor. One hospital did have L&D operating rooms located on a separate floor. Antepartum or postpartum rooms were frequently located on a separate floor.

### Unit and room square footage

**Fig 1:**
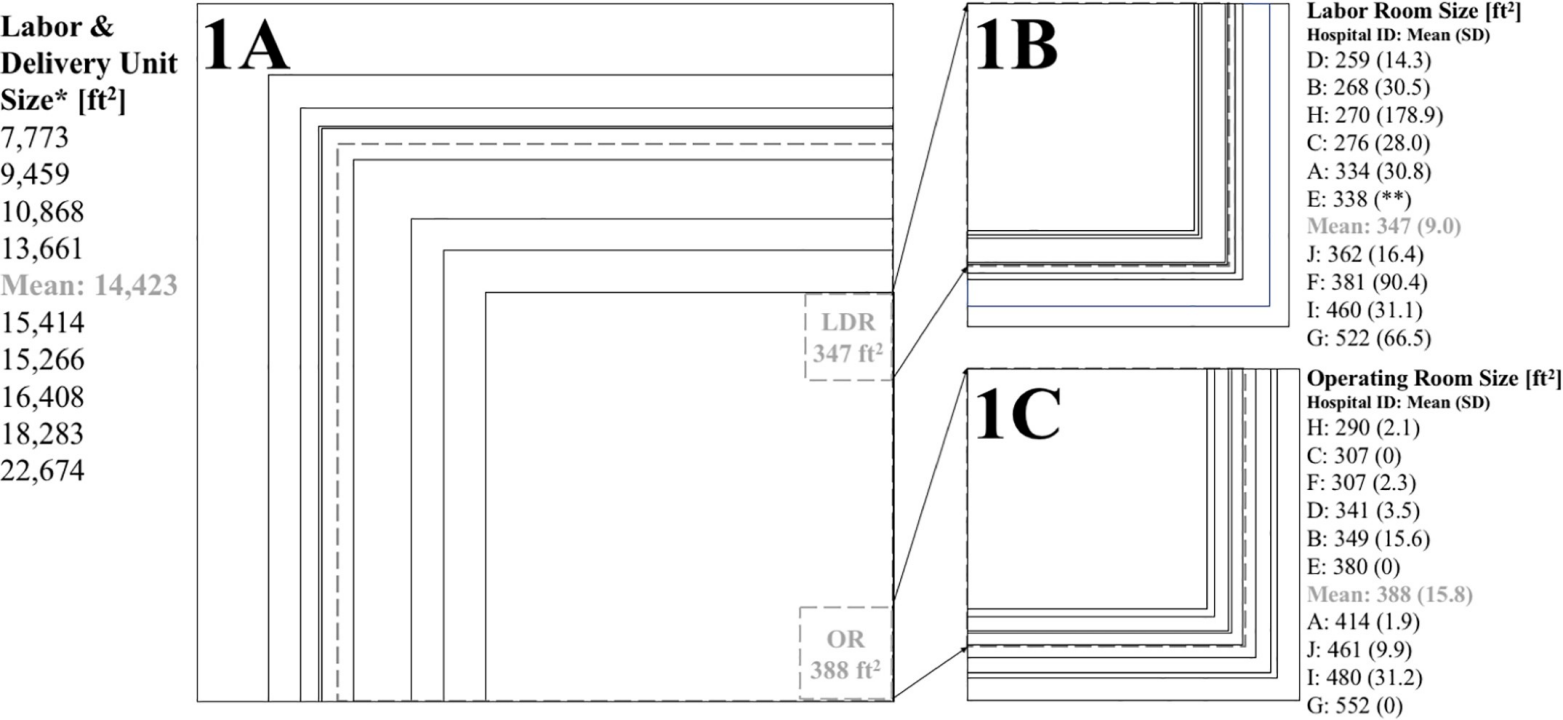
Labor unit, labor room, and operating room size for each hospital. 1A. Labor unit size comparison overlay. Each solid line box represents scaled size (ft^2^) of a labor unit, including all labor rooms, operating rooms, storage areas, hallways, provider workspaces, and patient and family areas. Provider call rooms and locker rooms were not included. The dashed line box represents the mean square footage of all units. Mean labor room and operating room shown to scale in grey for reference. Nine labor units included because, for hospital E, no blueprints available to measure total unit square footage. *Hospitals listed in ascending order by unit square footage (independent of randomized hospital identification) to protect identity. 1B. Labor room size comparison overlay. Each solid line box represents the mean labor room size (ft^2^) within a labor unit. The dashed line represents the mean labor room size across units. **No blueprints available to obtain standard deviation. 1C. Operating room size comparison overlay. Each solid line box represents the mean operating room size (ft^2^) within a labor unit. The dashed line represents the mean operating room size across units.

The approximate square footage of all labor rooms and operating rooms and the total unit was calculated. Total unit square footage included labor rooms, operating rooms, hallways, storage rooms, waiting rooms, triage areas, bathrooms, and utility rooms. Locker/changing rooms and call/sleep rooms were not included in square footage calculations. None of the units had completely separate areas for L&D triage or urgent care. For one hospital, we were unable to accurately report the total unit square footage because blueprints were not available. The distribution of unit and room sizes is shown in Fig 1. The total unit square footage ranged from 7,773 ft^2^ to 22,674 ft^2^ (median 15,266 ft^2^, mean 14,423 ft^2^, *SD* 90 ft^2^). The labor rooms ranged in size from 246 ft^2^ to 686 ft^2^ (median 338 ft^2^, mean 349 ft^2^, *SD* 90 ft^2^). The operating rooms ranged from 288 ft^2^ to 552 ft^2^ (median 396 ft^2^, mean 393 ft^2^, *SD* 81 ft^2^).

### Room and configuration count

**Fig 2:**
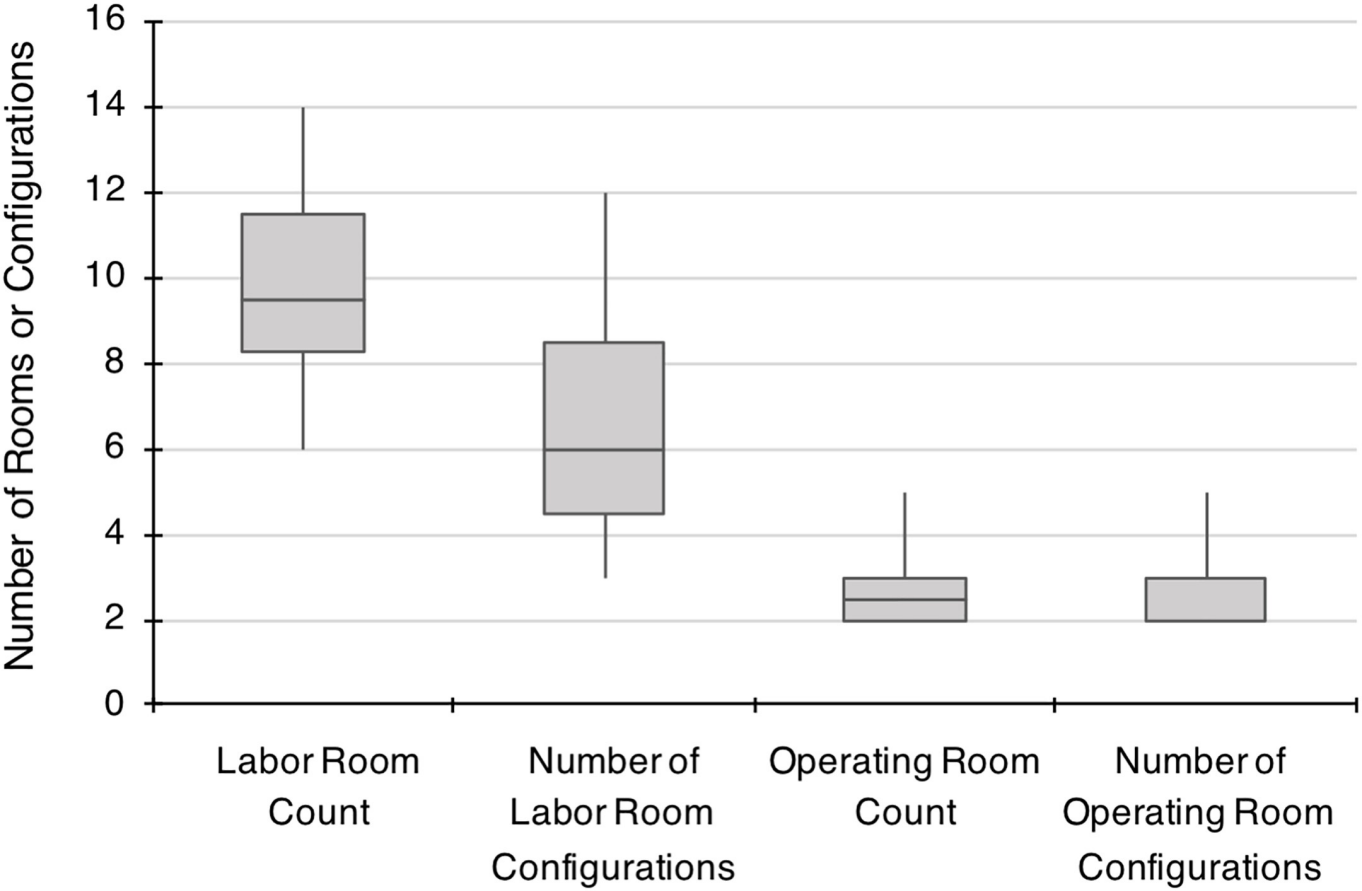
Distribution of count and number of configurations of labor rooms and operating rooms. Box and whisker plots represent median, first and third quartiles, and range for labor room and operating room counts and configurations across ten L&D units. Criteria for a different room configuration included different shape, equipment layout, entry location, or mirror image configuration.

Each unit was evaluated for the number of labor rooms and operating rooms, as well as the number of configurations of each. Configuration varied between the same room types, either labor room or operating room, within the same unit. For example, in one hospital, there was a different configuration for approximately every five labor rooms. By comparison, we observed several hospitals where there was a different configuration for every labor room. Across the entire sample there was a different labor room configuration for every 1.45 labor rooms (*SD* 0.87). There was a different operating room configuration for every 1.03 operating rooms (*SD* 0.16). Criteria for a different room configuration included different shape, equipment layout, entry location, and mirror image configuration. The distribution of room counts and number of configurations is shown in Fig 2. None of the ten labor units had one standard configuration for operating rooms or labor rooms.

### Labor room and operating room distance

**Fig 3:**
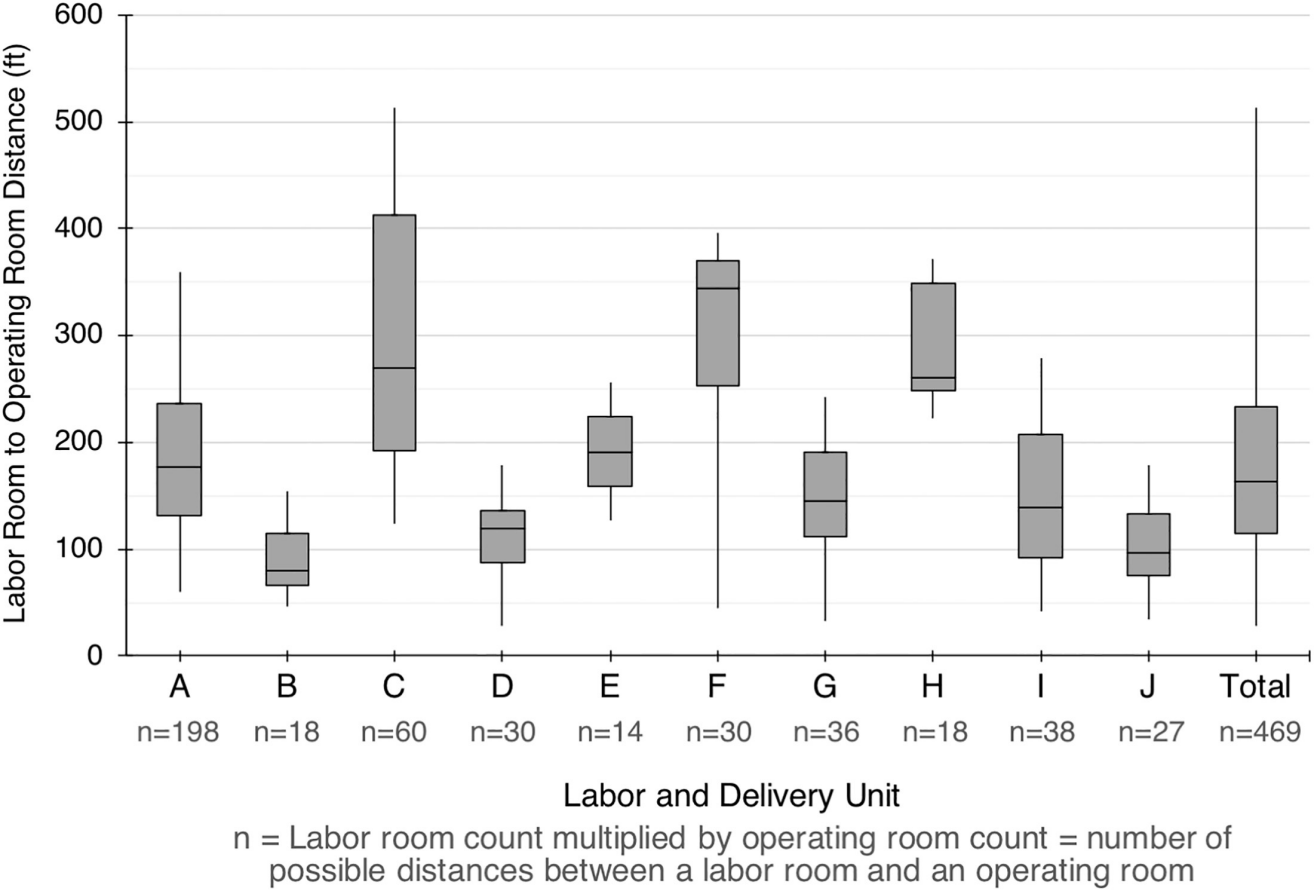
Distribution of labor room to operating room distances by labor unit. Box and whisker plots represent median, first and third quartiles, and range for the distances (ft) from each labor room to each operating room. n = Labor room count multiplied by operating room count. This number represents the number of possible distances required to transport a patient from a labor room to an operating room on L&D (i.e.: for an intrapartum Cesarean section or exam under anesthesia for postpartum hemorrhage).

To evaluate the transport distance for an intrapartum cesarean, the shortest distance between each labor room and operating room was obtained. The distribution of labor room to operating room distance for each unit is shown in Fig 3. There was a statistically significant difference between L&D units as determined by one-way ANOVA, *F* = 40.5, *p* < 0.01. Across all institutions, the labor room to operating room distance ranged from 28 ft to 396 ft (median 174 ft, mean 184, *SD* 83 ft). The mean labor room to operating room distance ranged from 88 ft to 309 ft.

### Labor and operating room equipment

An inventory of all equipment and the square footage it occupied in a representative labor room and operating room in each institution were measured. Findings showing common L&D equipment, their prevalence, and mean area are reported in Table 2. Despite the similarity of available equipment, the location of equipment in the room was highly variable.

**Table 2:** Inventory of equipment in labor and operating rooms.

	Labor Room			Operating Room		
	Item	Prevalence (%)	Area (ft^2^)	Item	Prevalence (%)	Area (ft^2^)
**Maternal**	Labor Bed	100	21.4	Bed	100	12
	Nightstand	80	3.3			
	Tray Table	80	4.4			
	Sitting/Rocking Chair	70	4.7			
	Shower	100	--			
	Bathtub	20	--			
**Fetal**	Infant Warmer	100	7.3	Infant Warmer	100	7.8
	Fetal Monitor Cabinet	100	4.9	Fetal Monitor Cabinet	100	3.5
				Neonatal Supply Cart	70	3.8
**Family**	Bed	60	18.7	Sitting Chair	100	1.9
	Sleeper Chair	50	7.8			
**Clinical Care**	Delivery Cart	90	4.9	Supply Cabinet	100	9.5
	IV Pole	90	3.2	Mayo Stand	100	2.5
	Computer Workstation	90	5.2	Surgical Table	100	8.1
	Sitting Stool	80	3.2	Prep Stand	80	2.8
	Wall Mounted Gloves	100	--	Suction Device	100	3.4
				Cautery Cart	100	3.2
				Basin Stand	80	2.3
				Standing Step	100	1.7
				IV Pole (x2)	100	3.2
				Forced Air Warmer	90	1.4
				Computer Workstation	90	5.3
				Sitting Stool	90	3.2
				Wall Mounted Gloves	100	--
				Rollerboard/Hovermat	80	--
				Anesthesia Machine	100	14.6
				Anesthesia Cart	100	8.3
				Anesthesia Computer	100	4.2
				Chair	100	2.8
				Videolaryngoscope	60	--
**Refuse**	Plain Trash	80	1.9	Biohazard	100	2.4
	Biohazard	90	2.0	Anesthesia Biohazard	80	1.6
	Linen	90	2.6	Linen	100	2.9
	Wall Sharps	100	0.5	Surgical Sharps	80	1.7
				Anesthesia Sharps	90	1.6
				Lap Sponge IV Pole	90	3.2
				Kick Bucket	80	0.9
		**Total Area = 96.0 ft**^**2**^			**Total Area = 119.8 ft**^**2**^	

### Statistical analysis

**Fig 4:**
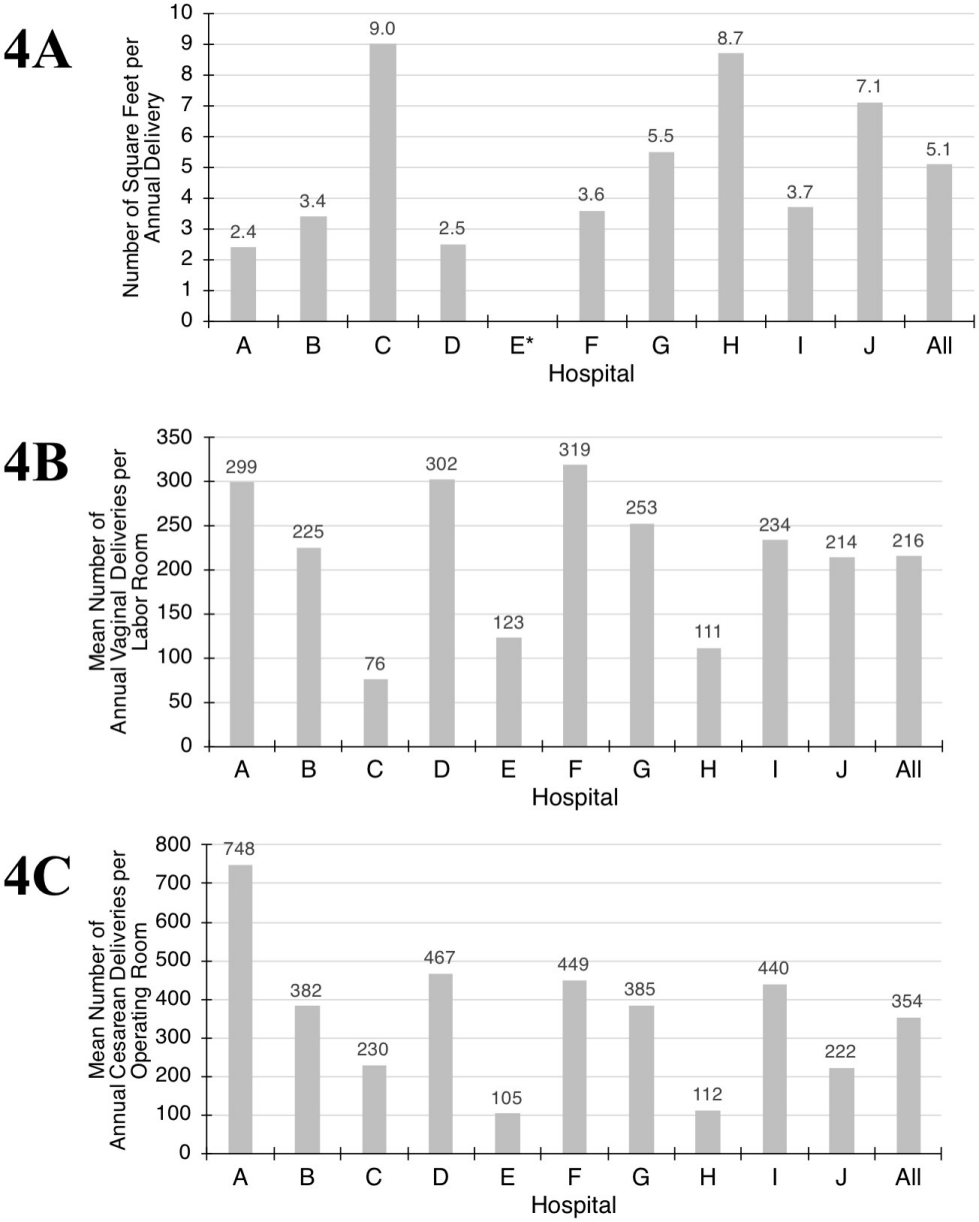
Unit, labor room and operating room delivery capacity. 4A. Ratio of labor unit square footage to annual delivery volume, or the number of square feet per annual delivery by hospital. *Hospital E excluded (unable to measure unit square footage). 4B. Ratio of annual vaginal deliveries to labor room count, or the mean number of vaginal deliveries in each labor room per year by hospital. 4C. Ratio of annual cesarean deliveries to operating room count, or the mean number of Cesarean deliveries in each operating room per year by hospital.

To analyze the range of capacity and utilization across labor units, we compared delivery volumes with unit square footage and room counts. The distributions are shown in Fig 4.

The ratios were highly variable: unit square footage: annual deliveries ranged from 2.4 to 9.0 (median 3.7, mean 5.1, *SD* 2.6), annual deliveries: labor room count ranged from 109 to 496 (median 327, mean 309, *SD* 123), annual deliveries: operating room count ranged from 446 to 1885 (mean 1164, median 1385, *SD* 437), annual vaginal deliveries: labor room count ranged from 76 to 319 (median 229, mean 319, *SD* 78), and annual cesarean deliveries: operating room count ranged from 105 to 748 (median 383, mean 354, *SD* 176).

To further analyze utilization within and across labor units, we constructed a graphic comparing quartiles for delivery volumes, delivery volume to room count ratios, delivery rates, and delivery rates to room count ratios (Fig 4). Clustering was more apparent among the delivery volume metrics than the delivery rate metrics. There was also clustering between cesarean delivery rate and NTSV cesarean rates.

Correlation analysis is shown in Table 3. We observed significant correlation between delivery volume and cesarean delivery rate (*r* = 0.88, *p* < 0.01) and annual deliveries and unit size (*r* = 0.77, *p* < 0.05). In other words, an increase in delivery volume was associated with a higher cesarean delivery rate and greater unit size. Cesarean section rate was positively correlated with labor and operating room counts; however, there was no significant association with 1) distances between labor rooms and operating rooms or 2) sizes of labor rooms and operating rooms.

**Table 3:** Summary of correlation coefficients (r) between design characteristics and delivery characteristics.

	Design Characteristics					
Delivery Characteristics	Unit Size (r)	LR Size (r)	OR Size (r)	LR Count (r)	OR Count (r)	LR to OR Distance (r)
Annual Deliveries	0.72*	0.05	0.08	0.75*	0.94**	-0.001
Vaginal Deliveries	0.72*	0.08	0.07	0.71*	0.93**	-0.02
Cesarean Deliveries	0.71*	0.02	0.08	0.79**	0.95**	0.02
NTSV Cesarean Deliveries	0.72*	0.03	0.05	0.78**	0.93**	0.03
Cesarean Rate	0.62	0.04	0.14	0.86**	0.80**	-0.06
NTSV Cesarean Rate	0.27	0.001	-0.33	0.77**	0.62	-0.30

LR = Labor Room, OR = Operating Room, NTSV = Nulliparous Term Singleton Vertex.

* = p<0.05,

** = p<0.01

### Scale rendering

**Fig 5:**
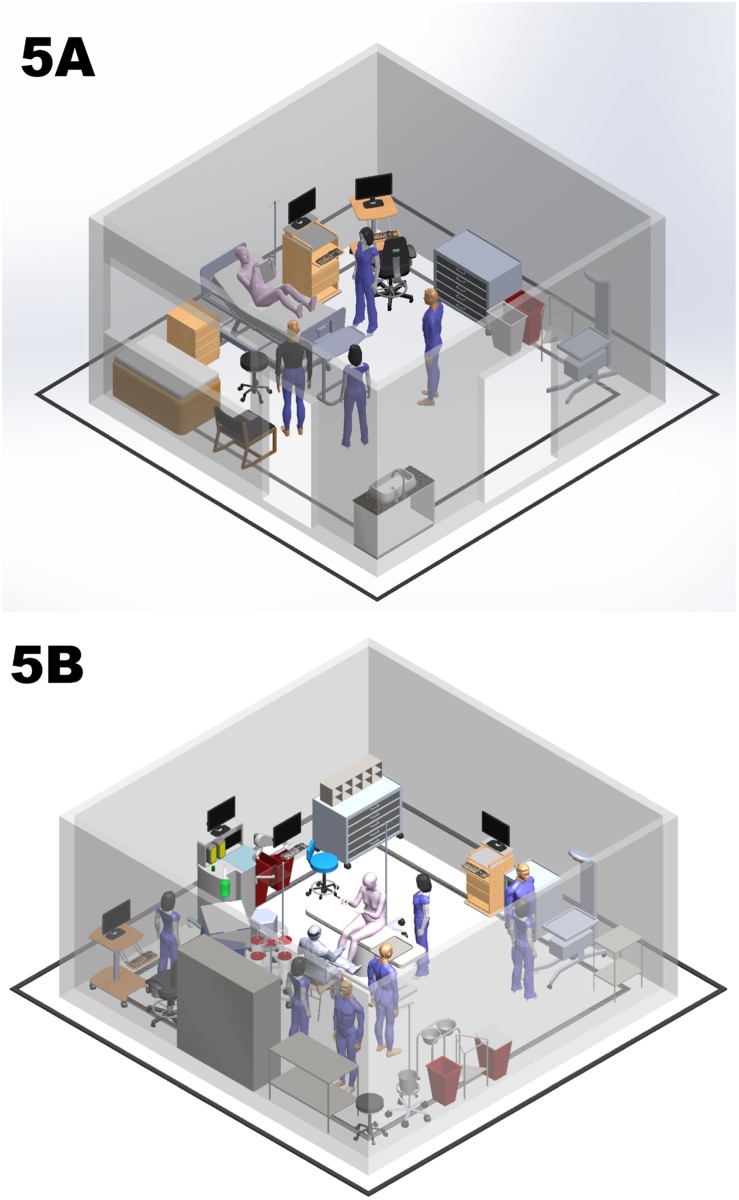
Scale renderings of labor room (5A) and operating room (5B). Room size, common equipment and approximate number of providers shown to scale. Flat black squares represent hospitals with lowest and highest mean room square footage. Upright walls represent mean square footage of all hospitals.

Fig 5A is a visual representation of measured variation of labor room size with overlay of common equipment and providers shown to scale. Fig 5B is the corresponding overlay for operating room size. Labor rooms and operating rooms with an overall mean or largest mean room square footage have more working space and less crowding.

## Discussion

We studied physical space of ten existing L&D units across California, representing a wide range of delivery volumes, unit ages, and mix of private and public institutions. Our primary finding is significant heterogeneity within and among units in terms of unit size, unit configuration, room size, room count, room configuration, and labor room to operating room proximity. Our results demonstrate significant homogeneity of equipment in labor rooms and operating rooms. The clinical significance of heterogeneity of space but homogeneity of equipment cannot be determined from our study. Specifically, while we explored L&D unit space in relation to total and NTSV cesarean rates, this correlation is not causation. Cesarean rates and other clinical outcomes may be influenced by many other factors including obstetric and neonatal level of care, patient acuity, maternal socioeconomic status, staffing, location, and cultural differences.

Analyzing physical measurements with CMQCC hospital delivery data showed correlation between unit size, room counts, and delivery volume. Logically, units with more deliveries will require more space, however, it is unclear whether units were designed for their current capacity or have evolved to accommodate it. The uncertainty is especially true in L&D units that were partly remodeled at different times. In units composed of sections that had been remodeled at different times, each subsequent remodel was built up to codes that existed at the time of construction. In our complementary paper [PONE-D-18-14892], we discuss how staff at some units retrofitted older spaces to accommodate delivery volumes.

The size of a unit and the spaces within a unit needed to meet specific clinical needs is unknown. While we understand obstetric and neonatal care is extremely dynamic and complex because of variations in time of day, time of year, and patient needs, the heterogeneity we found just within the ten units we reviewed was significant. Our results indicate there is nearly a four-fold difference in total unit size relative to delivery volume. We measured a four-fold difference in labor unit usage based on labor room count and volume, and there is a seven-fold difference in operating room usage based on operating room count and clinical volume. This study did not directly investigate causes for this variance in unit size and room usage.

Developing more standardized space guidelines is important. Space design is paramount to safety and quality of care while the space itself is a resource that is limited and expensive. The variation in design among units we measured revealed many areas for potential standardization in L&D unit design.

One area of potential improvements in L&D design standardization may involve planning labor unit size based on current or projected future delivery volume. This planning may achieve balance between a unit that is too small (frequently over capacity and unable to meet clinical needs) and a unit that is too large (frequently under capacity, greater building and overhead costs, and longer transit distances for clinical care). A second potential for standardization is labor and operating room counts needed for clinical volume. A unit with too few labor rooms will frequently be over capacity and have to choose between going on diversion or having women deliver in hallways, triage rooms, or operating rooms. Conversely, a unit with too many labor rooms will have wasted space and be unnecessarily large. An appropriate operating room count will keep elective cases on schedule while also accommodating urgent or emergent operating room transfers.

The third potential for standardization is room size and equipment. Our observation of relatively ubiquitous equipment in labor and operating rooms provides the potential to approximate necessary square footage footprint. For example, we found common labor room equipment occupied approximately 96 ft^2^ and estimate that each provider and family member will require additional space [34]. For example, at the time of delivery, you can estimate anthropometric space for two obstetricians, two nurses, and two family members in the labor room. Combining these estimates with local building code, other established guidelines, and further human factors research will provide an estimate for the size needed to comfortably and safely accommodate end-users.

Creating a gold standard for L&D units of course will require more than analyzing physical measurements with delivery volume and clinical outcomes of vaginal and cesarean delivery. Further research would need to control for confounding variables like maternal level of care and socioeconomic status. Surveys could help determine patient and provider satisfaction within existing units, and space planning teams could simulate and prototype new unit designs based on projected delivery volumes. Our investigative, design-centered approach has a number of benefits. We conceived of this project in an open, curious way that allowed us to ask many types of questions about these units because we truly did not know how L&D unit size, configuration and design varied across hospitals. Our approach was investigative because these units were operational and we observed these spaces as they were being used today. Visiting a mixture of private and public L&D units with high and low annual delivery volumes, we were able to correlate our measurements with current delivery volumes and limited clinical outcomes at these units. Our multidisciplinary team was composed of subspecialties (i.e., design, engineering, medicine) who rarely work together and brought clinical and non-clinical perspectives to this observational study.

This study design has several limitations. Our detailed data collection process limited the feasible sample size in this study. As a result, selection bias may exist as only ten hospitals were included. The ten hospitals measured were predominantly from urban and suburban areas in one state. Our sample did not include any rural L&D units. While California building codes set the minimum size at the time of construction, our approach was unable to measure some of the largest determinants of size and design on L&D units. We were neither privy to the facility budgets of these units at the time of construction nor their current operational costs, two factors which undoubtedly factor in to space and design.

The most significant limitation of our study is that it does not link a unit’s space, configuration, and equipment usage to other important factors such as staffing requirements, in-depth clinical outcomes, or maternal/neonatal level of care. This pilot study identified opportunities for performing formal needs assessment as well as running simulations to prototype different room configurations and unit layouts.

Further research could refine space estimates and determine generalizability across a broader number of units and locations. Subsequent investigations could help determine better estimates for ideal L&D unit size, configuration, and utilization that 1) ensure high quality of care, 2) optimize maternal and neonatal healthcare cost, and 3) allow for some variance based on individual hospital needs and constraints. The field may also benefit from knowing the proximity of L&D to other related locations throughout the hospital (i.e. NICU, Adult Main Operating Room, Pediatric Main Operating Rooms, Interventional Radiology, Emergency Room, Radiology). Additional research could also take into account construction costs and other variables.

Once opened, L&D units will need to function with the same size and footprint for decades. Building ideal design standards may be useful for units that are being newly constructed, remodeled, or retrofitted. Clinicians providing care and patients receiving it should be actively involved in the design process to maximize safety, efficiency, and experience of care. A well-designed unit may also have positive implications on patient and provider recruitment, retention, and satisfaction. Increased interest in physical design will help define optimal designs and configurations, bridging the gap between current design and building practices and the clinical point of care.

## References

[1] International Civil Aviation Organization. Standards and Recommended Practices. [cited 2018 September]. [Internet]. http://www.icao.int/safety/safetymanagement/pages/sarps.aspx.

[2] Arriaga AF, Bader AM, Wong JM, Lipsitz SR, Berry WR, Ziewacz JE, et al Simulation-based trial of surgical-crisis checklists. N Engl J Med. 2013;368(3):246–53. 10.1056/NEJMsa1204720 . 23323901

[3] Rhodes A, Evans LE, Alhazzani W, Levy MM, Antonelli M, Ferrer R, et al Surviving Sepsis Campaign: International Guidelines for Management of Sepsis and Septic Shock: 2016. Crit Care Med. 2017 10.1097/CCM.0000000000002255 . 28098591

[4] Centers for Disease Control and Prevention. Severe Maternal Morbidity in the United States. 2017 [cited 2018 September]. [Internet]. https://www.cdc.gov/reproductivehealth/maternalinfanthealth/severematernalmorbidity.html.

[5] World Health Organization and Human Reproduction Programme. WHO statement on caesarean section rates. 2015 [cited 2018 September]. [Internet]. http://www.who.int/reproductivehealth/publications/maternal_perinatal_health/cs-statement/en/.

[6] Lipman S, Cohen S, Einav S, Jeejeebhoy F, Mhyre JM, Morrison LJ, et al The Society for Obstetric Anesthesia and Perinatology consensus statement on the management of cardiac arrest in pregnancy. Anesth Analg. 2014;118(5):1003–16. 10.1213/ANE.0000000000000171 . 24781570

[7] California Maternal Quality Care Collaborative. CMQCC Maternal Quality Improvement Toolkits. [cited 2018 September]. [Internet]. https://www.cmqcc.org/resources-tool-kits/toolkits.

[8] Jeejeebhoy FM, Zelop CM, Lipman S, Carvalho B, Joglar J, Mhyre JM, et al Cardiac Arrest in Pregnancy: A Scientific Statement From the American Heart Association. Circulation. 2015;132(18):1747–73. 10.1161/CIR.0000000000000300 . 26443610

[9] Council on Patient Safety in Women’s Health Care. National Partnership for Maternal Safety. [cited 2018 September]. [Internet]. http://safehealthcareforeverywoman.org/patient-safety-bundles/.

[10] Committee on Equipment and Facilities of the American Society of Anesthesiologists. Operating Room Design Manual. 2010–2012 [cited 2018 September]. [Internet]. http://www.asahq.org/resources/resources-from-asa-committees/operating-room-design-manual.

[11] Kohn LT, Corrigan J, Donaldson MS. To err is human: building a safer health system. Washington, D.C.: National Academy Press; 2000 xxi, 287 p. p. 25077248

[12] Leape LL, Lawthers AG, Brennan TA, Johnson WG. Preventing medical injury. QRB Qual Rev Bull. 1993;19(5):144–9. . 8332330 10.1016/s0097-5990(16)30608-x

[13] Kirkpatrick DH, Burkman RT. Does standardization of care through clinical guidelines improve outcomes and reduce medical liability? Obstet Gynecol. 2010;116(5):1022–6. 10.1097/AOG.0b013e3181f97c62 . 20966684

[14] Milland M, Christoffersen JK, Hedegaard M. The size of the labor wards: is bigger better when it comes to patient safety? Acta Obstet Gynecol Scand. 2013;92(11):1271–6. 10.1111/aogs.12229 . 24015949

[15] The American Institute of Architects (AIA) Academy of Architecture for Health, Facilities Guidelines Institute (FGI). Guidelines for design and construction of health care facilities. Washington, DC: American Institute of Architects.

[16] AIA issues new Guidelines for the Design and Construction of Healthcare Facilities. Healthc Hazard Manage Monit. 2006;20(3):1–6. . 17144636

[17] Facilities Guidelines Institute, United States. Department of Health and Human Services, AIA Academy of Architecture for Health Guidelines for design and construction of health care facilities. 2006 ed Washington, DC: American Institute of Architects; 2006 325 p p.

[18] Facilities Guidelines Institute, United States. Department of Health and Human Services, AIA Academy of Architecture for Health. Guidelines for design and construction of hospitals and outpatient facilities. 2014 ed2014.

[19] Sprague JG. Development of guidelines for design and construction of hospitals and health care facilities. World Hosp Health Serv. 2003;39(3):35–8, 43, 5 . 14963892

[20] Rashid M. A decade of adult intensive care unit design: a study of the physical design features of the best-practice examples. Crit Care Nurs Q. 2006;29(4):282–311. . 17063097 10.1097/00002727-200610000-00003

[21] Thompson DR, Hamilton DK, Cadenhead CD, Swoboda SM, Schwindel SM, Anderson DC, et al Guidelines for intensive care unit design. Crit Care Med. 2012;40(5):1586–600. 10.1097/CCM.0b013e3182413bb2 . 22511137

[22] Guidelines for intensive care unit design. Guidelines/Practice Parameters Committee of the American College of Critical Care Medicine, Society of Critical Care Medicine. Crit Care Med. 1995;23(3):582–8. . 7874913

[23] Gulwadi GB, Joseph A, Keller AB. Exploring the impact of the physical environment on patient outcomes in ambulatory care settings. HERD. 2009;2(2):21–41. . 21161928 10.1177/193758670900200203

[24] O’Connor M, O’Brien A, Bloomer M, Morphett J, Peters L, Hall H, et al The environment of inpatient healthcare delivery and its influence on the outcome of care. HERD. 2012;6(1):104–16. . 23224845 10.1177/193758671200600106

[25] Co Pathologists. Guidelines on laboratory construction and design. Malays J Pathol. 2005;27(1):63–7. . 16676696

[26] Welch SJ. Using data to drive emergency department design: a metasynthesis. HERD. 2012;5(3):26–45. . 23002567 10.1177/193758671200500305

[27] MASS Design Group. The Impact of Design on Clinical Care in Childbirth. 2017 [cited 2018 September]. [Internet]. https://massdesigngroup.org/work/research/impact-design-clinical-care-childbirth.

[28] California Department of Public Health, Maternal Child and Adolescent Health Division. The California Pregnancy-Associated Mortality Review. Report from 2002 and 2003 Maternal Death Reviews. 2011 [cited 2018 September]. [Internet]. https://www.cmqcc.org/research/ca-pamr-maternal-mortality-review.

[29] Main EK, McCain CL, Morton CH, Holtby S, Lawton ES. Pregnancy-related mortality in California: causes, characteristics, and improvement opportunities. Obstet Gynecol. 2015;125(4):938–47. 10.1097/AOG.0000000000000746 . 25751214

[30] Menard MK, Kilpatrick S, Saade G, Hollier LM, Joseph GF, Barfield W, et al Obstetric Care Consensus No. 2: Levels of maternal care. Obstet Gynecol. 2015;125(2):502–15. 10.1097/01.AOG.0000460770.99574.9f . 25611640

[31] Newborn AAoPCoF. Levels of Neonatal Care. Pediatrics. 2012;130(3):587–97. 10.1542/peds.2012-1999 . 22926177

[32] National Center for Education Statistics. Search for Public School Districts: Locale. [cited 2018 September]. https://nces.ed.gov/ccd/districtsearch/.

[33] California Department of Public Health. Health Facilities Consumer Information Service (HFCIS). [cited 2018 September]. http://hfcis.cdph.ca.gov/default.aspx.

[34] Panero J, Zelnik M. Human dimension & interior space: a source book of design reference standards. New York: Whitney Library of Design; 1979 320 p. p.

